# RAIM and Failure Mode Slope: Effects of Increased Number of Measurements and Number of Faults

**DOI:** 10.3390/s23104947

**Published:** 2023-05-21

**Authors:** Jean-Bernard Uwineza, Jay A. Farrell

**Affiliations:** Department of Electrical and Computer Engineering, University of California, Riverside, CA 92521, USA; buwineza@ece.ucr.edu

**Keywords:** navigation, fault detection, RAIM, GNSS

## Abstract

This article provides a comprehensive analysis of the impact of the increasing number of measurements and the possible increase in the number of faults in multi-constellation Global Navigation Satellite System (GNSS) Receiver Autonomous Integrity Monitoring (RAIM). Residual-based fault detection and integrity monitoring techniques are ubiquitous in linear over-determined sensing systems. An important application is RAIM, as used in multi-constellation GNSS-based positioning. This is a field in which the number of measurements, *m*, available per epoch is rapidly increasing due to new satellite systems and modernization. Spoofing, multipath, and non-line of sight signals could potentially affect a large number of these signals. This article fully characterizes the impact of measurement faults on the estimation (i.e., position) error, the residual, and their ratio (i.e., the failure mode slope) by analyzing the range space of the measurement matrix and its orthogonal complement. For any fault scenario affecting *h* measurements, the eigenvalue problem that defines the worst-case fault is expressed and analyzed in terms of these orthogonal subspaces, which enables further analysis. For h>(m−n), where *n* is the number of estimated variables, it is known that there always exist faults that are undetectable from the residual vector, yielding an infinite value for the failure mode slope. This article uses the range space and its complement to explain: (1) why, for fixed *h* and *n*, the failure mode slope decreases with *m*; (2) why, for a fixed *n* and *m*, the failure mode slope increases toward infinity as *h* increases; (3) why a failure mode slope can become infinite for h≤(m−n). A set of examples demonstrate the results of the paper.

## 1. Introduction

Multi-constellation Global Navigation Satellite Systems (GNSSs) have reached a maturity level where they are routinely relied upon as critical positioning systems that require a high degree of integrity [1,2]. The integrity of a positioning system is a measure of how much trust can be allocated to the correctness of the position solution [3]. This measure includes the ability of that system to be monitored (by itself or otherwise) and to provide timely warnings to the user when the system should not be used for positioning. Integrity monitoring, thus, requires the system to detect faulty measurements before they cause out-of-specification performance [4].

Receiver Autonomous Integrity Monitoring (RAIM) is one well-established method of ensuring the consistency of the positioning solution and assessing the integrity risk posed by available measurements [4,5,6]. Integrity risk is defined as the probability of undetected faults causing unacceptably large positioning errors [7]. Current implementations of residual-based RAIM (RB RAIM) are designed to detect faulty measurements and to evaluate the safety risk posed by possibly undetected faults (see Section 23.7 in [8]).

Integrity monitoring is applicable to a wide array of sensors that provide redundant measurements. Early approaches to RAIM emphasized its use to supplement various modes of aerospace navigation [9,10,11] or the ability of RAIM to enhance satellite-based augmentation services [12], such as the Wide Area Augmentation System (WAAS) [13]. The research in [13,14] proposed an implementation of weighted RAIM to compute Vertical Protection Levels (VPLs) for precision approach and landing supported by information from WAAS. Notable is that all the above methods assumed only single-measurement faults. This was justified by the assumption that measurement faults were caused by partial or complete satellite failure and that the likelihood of more than one satellite failing simultaneously is small.

Multi-measurement fault scenarios can be computationally challenging. In a scenario where the total number of measurements is denoted by *m* and the number of faulty measurements is denoted by *h*, the number of combinations of sensor faults that must be considered is mh, which grows rapidly with both *m* and *h*.

The growing availability of multiple GNSS constellations with multi-frequency measurements available per system means that the number of measurements available per epoch is rapidly increasing; therefore, *m* entering the 50–100 range is not unreasonable even in low-cost receivers [15,16,17,18].

In emerging applications, such as urban automotive navigation, healthy satellites could have faulty measurements (i.e., out-of-specification range errors) due to non-line-of-sight signals [19,20], strong multipath [21,22], ionospheric effects [23,24,25], and other environmental factors [26]. In low-latitude regions, ionospheric scintillation can be more pronounced [27], and persistent satellite oscillator anomalies resembling ionospheric scintillation have been recently reported to affect multiple GPS satellites [28,29]. Spoofing is an important threat to GNSS, which is achieved by simultaneously altering all satellite measurements in a targeted region [30,31,32,33]. These examples demonstrate that multi-measurement faults (i.e., h≫1) are a reasonable possibility that cannot be ignored.

In such multi-fault hypotheses, the fault direction is unknown; therefore, an upper bound on the integrity risk is derived for the worst-case fault direction in each *h*-fault scenario, using the worst-case failure mode slope [7,34]. This slope is defined as the largest ratio of the fault-induced estimation error to the fault-induced residual [34]. The failure mode slope is infinite when a fault is undetectable from the residual vector. For example, when the fault is in the range space of the measurement matrix, it is undetectable and the failure mode slope is infinite (see p. 110 in [10]). Such a fault can always be found when h>(m−n), where *n* is the dimension of the vector that is being estimated. Single (i.e., h=1) and double measurement (i.e., h=2) fault scenarios are considered in, e.g., refs. [13,34], respectively. Multi-measurement fault scenarios (1≤h) are considered in [7,35], where the worst-case fault vector was determined by solving an eigenvalue problem. The worst-case slope is the maximum eigenvalue and the worst-case fault direction corresponds to its eigenvector. The analysis of [7] considers only one component of the state vector, which would be important in applications such as the vertical error in precision aircraft landing.

However, in other applications, such as land and sea navigation, there is interest in how multiple faults affect vector quantities, i.e., the horizontal position [36,37].

This paper studies how the RB RAIM failure mode slope changes as a function of the number of measurements, *m*, and the number of faulty measurements, *h*. The analysis uses the SVD as a tool: (1) to explicitly characterize the fault-induced estimation error and the fault-induced residual; (2) to find and characterize the linear space of faults that affect the residual, but not the estimate; (3) to find and characterize the linear space of (undetectable) faults that affect the estimate, but not the residual; and (4) to provide an analytic expression for the worst-case fault direction for all 1≤h≤m. In addition, the expression that results from the SVD approach provides additional physical insight into the solution. In the case where h>(m−n), this article provides an orthogonal basis for the (m−n) dimensional linear space of faults that have infinite failure mode slope and are, hence, undetectable by residual-based detectors. Finally, this paper shows that, even in the case that h≤(m−n), it is possible to have an infinite failure mode slope and provides analytic expressions that determine when this is the case. The examples in Section 10.4 demonstrate the main points of the article, including an example of undetectable faults when h=m−n.

This paper is organized as follows: Section 2 introduces the measurement model, including assumptions and models of the noise and fault. Section 3 defines the notation and symbols adopted. Section 4 uses the SVD decomposition of the measurement matrix to analyze the effects of noise and faults on the estimation error and residual. Section 5 reviews concepts related to integrity risk, while Section 6 reviews the concept of the failure mode slope. Section 7 presents new results on the best and worst-case fault direction vectors for general fault scenarios, showing them to be intrinsically related to certain singular vectors of the measurement matrix. An analytical expression for the upper bound on the estimation error is also provided. Section 8 analyzes worst-case fault modes for different fault scenarios, beginning with single-measurement faults, then double-measurement faults, and, ultimately, multi-measurement faults. Section 9 discusses the generalization of the approach when only subspaces of the estimated state are of interest. Section 10 presents and discusses GNSS examples demonstrating the results herein. Section 11 provides concluding remarks.

## 2. Problem Formulation

Consider the following over-determined linear measurement model, which applies to multi-constellation GNSS:

(1) y=Hx+η+f,

where $y\in R^{m\times 1}$ is the measurement vector; $H\in R^{m\times n}$ is the measurement matrix, with m>n and rank(H)=n; $x\in R^{n}$ is a vector to be estimated; $\eta \in R^{m}$ is measurement noise that is assumed to be Gaussian with $\eta ∼N(0,C_{\eta })$, where $C_{\eta }=\sigma_{\eta }^{2}\;I$; and $f\in R^{m}$ is the fault (sometimes also referred to as the bias or failure). The fault f is defined as:

(2) $$f≜\mu \;\vec{f},$$

where μ∈R is the magnitude of the fault, and the unit vector $\vec{f}$ is the fault direction. In the absence of a fault, μ=0.

This papers investigates the impact of f on the estimation error and the fault detector, within the context of evaluating Hazardously Misleading Information (HMI). This paper uses the term *fault scenario* to mean a combination of faults affecting *h* measurements (i.e., the vector $\vec{f}$ has *h* non-zero components). The *failure mode* is defined to be a specific combination of measurements within a fault scenario. Note that there are Mh=mh different failure modes within each fault scenario. For each failure mode, one of the goals of this paper is to explicitly define the worst-case fault direction and its failure-mode slope.

In many applications, the analyst is only interested in a rotated subvector of x. Such situations are easily addressed within the context of this article by defining z=Mx and applying the methods herein to the vector z instead of x. In this context, M combines a rotation and projection matrix. For example, in an automotive application, the horizontal position error is usually of primary interest. Assume that GNSS provides an estimate of $x={\left[x,\;y,\;z,\;b\right]}^{⊤}\in R^{4}$, where x,y,z are the position coordinates in an Earth-Centered Earth-Fixed (ECEF) reference frame, and *b* is the receiver clock bias. Then, $M\in R^{2\times 4}$ can be defined as:

(3) $$\begin{array}{c} M≜\left[\begin{array}{c} I_{2\times 2}|\;0_{2\times 2} \end{array}\right]\;{}_{E}^{T}R\;, \end{array}$$

where ${}_{E}^{T}R\in R^{4\times 4}$ is a rotation matrix from ECEF to tangent plane [38]. This definition of M transforms x from ECEF to tangent plane and only retains the horizontal position components, removing the vertical position and the clock bias components.

In the analysis that follows, the derivations will be performed on the general case, estimating the full estimation vector x. Later in the paper, the horizontal position special case will be considered.

## 3. Notation

Different equality symbols will be used to distinguish between definitions, computations, and theoretical models used for analysis. The symbol ‘≜’ indicates a *definition* of a specific model or quantity. The symbol ‘≐’ indicates that the quantity on the left-hand side can computed from the quantities on the right-hand side. The symbol ‘=’ indicates that an equation is a theoretical model, not a definition or a computation used in implementations. Such models are used for analysis and physical interpretation of quantities.

The analysis of this article will use the singular value decomposition (SVD). The SVD of a matrix $H\in R^{m\times n}$ is

(4) $$H≜\left[U_{1},U_{2}\right]\left[\begin{array}{c} \Sigma \\ 0 \end{array}\right]V^{⊤},$$

where $U=\left[U_{1},U_{2}\right]\in R^{m\times m}$ is a unitary matrix with two orthogonal submatrices $U_{1}=\left[u_{1}^{1},\cdots ,u_{1}^{n}\right]\in R^{m\times n}$ and $U_{2}=\left[u_{2}^{1},\cdots ,u_{2}^{m-n}\right]\in R^{m\times (m-n)}$; $\Sigma \in R^{n\times n}$ is positive definite and diagonal, where the diagonal elements $\alpha_{1}\geq \alpha_{2}\geq \ldots \geq \alpha_{n}>0$ are the singular values of H; and $V\in R^{n\times n}$ is unitary. These matrices have interpretations that are useful for the analysis: V is an orthonormal basis for the domain of H; $U_{1}$ is an orthonormal basis for the *n* dimensional linear space that is the range of H; and $U_{2}$ is an orthonormal basis for the (m−n) dimensional linear space that is orthogonal to the range of H.

The term *eigenvalue problem* will refer to the constrained quadratic optimization problem of the form:

(5) $$\begin{array}{cc} x^{*} & =\arg\max_{{∥x∥}^{2}=1}x^{⊤}\;A\;x=\arg\max_{x\neq 0}\frac{x^{⊤}\;A\;x}{x^{⊤}x}, \end{array}$$

where $A\in R^{n\times n}$ is a real symmetric matrix. The solution $x^{*}\in R^{n}$ is the eigenvector corresponding to the largest eigenvalue of A [39]. That eigenvalue is $\max_{{∥x∥}^{2}=1}x^{⊤}\;A\;x$.

When necessary, the actual and estimated values of a quantity are distinguished by *x* and $\hat{x}$, respectively. Vectors and matrices are written in boldface, with vectors in lowercase and matrices in uppercase. For example, $v\in R^{n}$ is an *n*-element vector and $V\in R^{n\times n}$ is an n×n matrix. Positive definiteness (i.e., $v^{⊤}V\;v>0,\;\forall v\neq 0$) and semi-definiteness (i.e., $v^{⊤}V\;v\geq 0,\;\forall v\neq 0$) of a matrix is indicated by V≻0 and V⪰0, respectively. The matrix $I_{n\times n}=I_{n}\in R^{n\times n}$ is the n×n identity matrix. The symbol $\vec{v}$ indicates the unit vector in the direction of vector v. The vector $e_{i}$ is the *i*-th column of I, which is the *i*-th standard basis vector of $R^{n}$, where i≤n. Throughout the article, tr(·) is the trace operator and ∥·∥ is the $L^{2}$ norm. For random vector ζ, its mean squared value will be denoted as ${∥\zeta ∥}_{M}^{2}=\;E\langle \zeta^{⊤}\zeta \rangle$. Other notations will be defined when used.

## 4. Analysis of the Estimation Error and Residual

For measurements described by Equation (1), the minimum-variance unbiased (MVU) estimate of x is computed as [40]:

(6) $$\begin{array}{cc} \;\;\;\;\;\;\hat{x} & ≐{\left(H^{⊤}C_{\eta }^{-1}H\right)}^{-1}H^{⊤}C_{\eta }^{-1}y \end{array}$$

(7) $$\begin{array}{cc} & ≐H^{*}\;y\;, \end{array}$$

where $H^{*}≜{\left(H^{⊤}C_{\eta }^{-1}H\right)}^{-1}H^{⊤}C_{\eta }^{-1}$. In the special case where $C_{\eta }=\sigma_{\eta }^{2}\;I$, this simplifies to $H^{*}≜{\left(H^{⊤}H\right)}^{-1}H^{⊤}$. This special case is the main focus of this article.

The analyses that follow use the SVD of H to analyze the impact of faults on the estimation of $\hat{x}$. Substituting Equation (4) into the definition of $H^{*}$ in Equation (7) yields $H^{*}=V\;\Sigma^{-1}\;U_{1}^{⊤}$, so that Equation (7) becomes:

(8) $$\begin{array}{cc} \hat{x} & ≐V\;\Sigma^{-1}\;U_{1}^{⊤}\;y. \end{array}$$

Equation (8) is derived in Equation (S5) of Section S1 in the Supplementary Materials [41].

### 4.1. Effects of Noise and Faults on the Estimate

To analyze the effect of the measurement noise η and fault f, substitute (1) into (7):

(9) $$\begin{array}{cc} \hat{x} & =\left({\left(H^{⊤}H\right)}^{-1}H^{⊤}\right)\left(H\;x+\eta +f\right)\\ \; & ={\left(H^{⊤}H\right)}^{-1}H^{⊤}H\;x+{\left(H^{⊤}H\right)}^{-1}H^{⊤}\left(\eta +f\right)\\ \; & =x+H^{*}\left(\eta +f\right). \end{array}$$

The estimate $\hat{x}$ is a Gaussian random variable with

$$E\langle \hat{x}\rangle =x+H^{*}\;f\;\mathrm{and}\;C_{x}=\mathrm{cov}\left(\hat{x}\right)=\sigma_{\eta }^{2}\;{\left(H^{⊤}H\right)}^{-1}=\sigma_{\eta }^{2}\;V\;\Sigma^{-2}\;V^{⊤}.$$

The estimation error defined as $\delta x=\hat{x}-x$ relates to the noise and fault vector as:

(10) $$\begin{array}{cc} \delta x & =H^{*}\left(\eta +f\right)=V\;\Sigma^{-1}\;U_{1}^{⊤}\eta +V\;\Sigma^{-1}\;U_{1}^{⊤}f\\ \; & =\delta x_{\eta }+\delta x_{f}. \end{array}$$

with noise-induced error $\delta x_{\eta }≜V\;\Sigma^{-1}\;U_{1}^{⊤}\eta$ and fault-induced bias $\delta x_{f}≜V\;\Sigma^{-1}\;U_{1}^{⊤}f$. The expected value and covariance of the estimation error are:

(11) $$\begin{array}{cc} \;\;\;\;\;\;E\langle \delta x\rangle & =H^{*}\;E\langle \eta +f\rangle =\delta x_{f}\;\mathrm{and} \end{array}$$

(12) $$\begin{array}{cc} \mathrm{cov}(\delta x) & =\sigma_{\eta }^{2}\;V\;\Sigma^{-2}\;V^{⊤}. \end{array}$$

The fault induces an unknown bias $\delta x_{f}$ on the estimate but does not affect its covariance, which remains $C_{x}$. The estimation error distribution is Gaussian:

(13) $$\delta x∼N(\delta x_{f},C_{x}).$$

Section S2 of the Supplementary Materials [41] shows that the mean-squared-error (MSE) of the estimate is:

(14) $$\begin{array}{cc} {∥\delta x∥}_{M}^{2} & ≜E\langle {\left(\delta x\right)}^{⊤}\;\;\left(\delta x\right)\rangle ={∥\delta x_{f}∥}^{2}+\kappa_{1}, \end{array}$$

where

(15) $$\begin{array}{cc} ∥\delta x_{f}{∥}^{2}={∥\Sigma^{-1}U_{1}^{⊤}f∥}^{2}\;\mathrm{and}\;\kappa_{1} & ≜\mathrm{tr}(C_{x}). \end{array}$$

Equation (14) shows that the fault and noise have independent effects on the norm of the estimation error.

The first term allows the evaluation of the effect of specific faults on the estimate, given any specific instances of the measurement matrix H and fault vector f. This will allow the analyst to find bounds on the estimation error under different fault scenarios.

It is convenient to rewrite the fault f using the orthogonal basis of $R^{m}$ defined by the columns of U:

(16) $$\begin{array}{c} f=\sum_{k=1}^{n}a_{k}\;u_{1}^{k}+\sum_{\ell =1}^{m-n}b_{\ell }\;u_{2}^{\ell }=U_{1}\;a+U_{2}\;b=\left[\begin{array}{cc} U_{1} & U_{2} \end{array}\right]\left[\begin{array}{c} a\\ b \end{array}\right], \end{array}$$

where $a\in R^{n}$ and $b\in R^{\left(m-n\right)}$ are coefficient vectors. Specifically,

(17) $$\begin{array}{cc} {\left[\begin{array}{cc} a & b \end{array}\right]}^{⊤} & ={\left[\begin{array}{cc} U_{1} & U_{2} \end{array}\right]}^{⊤}f. \end{array}$$

Substituting the orthogonal decomposition of (16) into (10) yields:

(18) $$\begin{array}{cc} \delta x\; & =\left(V\;\Sigma^{-1}U_{1}^{⊤}\right)\left(\left[\begin{array}{cc} U_{1} & U_{2} \end{array}\right]\left[\begin{array}{c} a\\ b \end{array}\right]+\eta \right)=V\;\Sigma^{-1}U_{1}^{⊤}U_{1}\;a+V\;\Sigma^{-1}U_{1}^{⊤}U_{2}\;b+\delta x_{\eta }\\ \; & =V\;\Sigma^{-1}a+\delta x_{\eta }. \end{array}$$

Equation (18) shows that $\delta x_{f}≜V\;\Sigma^{-1}\;U_{1}^{⊤}f=V\;\Sigma^{-1}a$, which has the following interpretation.

**Fact 1.**  *Any portion of a fault f that is within the span of the (m−n) dimensional linear space defined by the columns of $U_{2}$ has no effect on the estimate.* △

### 4.2. Effects of Noise and Faults on the Measurement Residual

The measurement residual is

(19) $$r≐y-\hat{y}=\left(I-P\right)\;y,$$

where $\hat{y}≐H\;\hat{x}≐P\;y$ with $P≜H\;H^{*}=U_{1}U_{1}^{⊤}\in R^{m\times m}$. The matrix P is the projection matrix onto the range space of H. It is symmetric, positive semi-definite, and idempotent with rank(P)=n. See Lemma S1 of Section S1 in the Supplementary Materials [41].

The effect of noise η and fault f on the measurement residual is analyzed by substituting (1) into (19):

(20) $$\begin{array}{cc} r & =\left(I-P\right)\left(H\;x+\eta +f\right)=\left(I-P\right)\left(\eta +f\right)\\ \; & =Q\;\eta +Q\;f. \end{array}$$

Equation (20) shows that $r=r_{f}+r_{\eta }$ where the fault-induced residual is $r_{f}=Q\;f$ and the noise-induced residual is $r_{\eta }=Q\;\eta .$ The matrix $Q≜\left(I-P\right)=U_{2}U_{2}^{⊤}\in R^{m\times m}$ is the projection matrix onto the complement of the range space of H. Therefore, QP=PQ=0 and QH=0. The properties of Q are discussed in Lemma S2 of Section S1 of the Supplementary Materials [41]. It is a symmetric, positive semi-definite, and idempotent matrix with rank(Q)=(m−n). Substituting Equation (16) into (20) yields

(21) $$\begin{array}{cc} r & =Q\;\eta +Q\;U_{2}\;b\\ \; & =Q\;\eta +U_{2}\;b. \end{array}$$

Equations (20) and (21) have the following interpretation.

**Fact 2.**  *Any portion of a fault f that is within the span of the n dimensional linear space defined by the columns of $U_{1}$ has no effect on the residual.* △

The expected value and covariance of r are:

(22) $$\begin{array}{cc} E\langle r\rangle & =Q\;f=U_{2}\;U_{2}^{⊤}\;f\;\mathrm{and}\;C_{r}≐\sigma_{\eta }^{2}\;Q=\sigma_{\eta }^{2}\;U_{2}\;U_{2}^{⊤}. \end{array}$$

The covariance matrix $C_{r}$ is only positive semi-definite, so not invertible [42]. The distribution of r is Gaussian:

(23) $$\begin{array}{c} r∼N(Q\;f,\;C_{r}). \end{array}$$

Section S3 of the Supplementary Materials [41] shows that the MSE of the residual is:

(24) $$\begin{array}{c} {∥r∥}_{M}^{2}={∥r_{f}∥}^{2}+\kappa_{2}, \end{array}$$

where

(25) $$\begin{array}{c} ∥r_{f}{∥}^{2}=∥U_{2}^{⊤}{\;f∥}^{2}\;\mathrm{and}\;\kappa_{2}≜{∥r_{\eta }∥}_{M}^{2}=\left(m-n\right)\;\sigma_{\eta }^{2}=\mathrm{tr}\left(C_{r}\right). \end{array}$$

The residual MSE defined in (24) is the sum of a fault-dependent component, $∥r_{f}{∥}^{2}$, and a noise-dependent component, $∥r_{\eta }{∥}_{M}^{2}$.

### 4.3. Fault Decisions

The residual-based test statistic is

(26) $$T_{r}≐r^{⊤}r={∥r^{⊤}r∥}^{2},$$

with faults declared when $T_{r}\geq T^{*}$. (Alternatively, RAIM methods can be defined based on a parity vector [7,10]. By the definition of a parity vector, it is the case that any parity vector must satisfy $p≜R^{⊤}\;U_{2}^{⊤}y$, where R is a unitary matrix. Therefore, $r=U_{2}\;R\;p$ and ∥r∥=∥p∥ because $U_{2}^{⊤}\;U_{2}=I$. Because ∥r∥=∥p∥, the two test statistics are completely equivalent. This article chooses to focus on the residual vector. All results herein extend directly to the parity vector.)

Based on Equation (19), the test statistic can also be computed as $T_{r}≐y^{⊤}Q\;y$. The procedures to select $T^{*}$ based on the probability of false alarm and continuity risk specifications are described in [13,43].

## 5. Integrity Risk Evaluation

Integrity risk evaluates the probability of having Hazardously Misleading Information (HMI) [8]. HMI refers to the case when the estimation error exceeds a predefined threshold referred to as *alarm limit L*, while the fault detector $T_{r}$ does not detect a fault. It is written as:

(27) $$\begin{array}{c} HMI≜\left(∥\delta x∥>L\right)\cap \left(T_{r}<T^{🟉}\right). \end{array}$$

The probability of HMI, P(HMI), is evaluated under different fault hypotheses $H_{h,i}$. The number of fault hypotheses for *m* measurements, of which *h* are fault-affected, is Mh=mh (i.e., the number of permutations of *m* items taken *h* at a time). Therefore, the integrity risk can then be expressed as:

(28) $$\begin{array}{cc} P(HMI) & =\sum_{h=1}^{m}P_{h}\left(HMI\right),\mathrm{where} \end{array}$$

(29) $$\begin{array}{cc} \;\;\;P_{h}\left(HMI\right) & =\sum_{i=0}^{M_{h}}P\left(HMI_{h,i}\;|\;H_{h,i}\right)\;P\left(H_{h,i}\right), \end{array}$$

where the possible $M_{h}$ hypotheses for each h=1,…,m are assumed mutually exclusive and jointly exhaustive.

Full evaluation of integrity risk requires evaluating each term in the summation of (28). However, this incurs a heavy computational cost when $M_{h}$ becomes large; therefore, some references employ hypothesis reduction approaches that only evaluate a subset of hypotheses, while accounting for the remaining hypotheses by assigning them a probability of occurrence [44]. For example, if a bound $P_{R}$ can be defined such that

$$P_{R}\geq \sum_{h=h^{🟉}+1}^{m}P_{h}\left(HMI\right),$$

then,

(30) $$\begin{array}{cc} P(HMI) & \leq \sum_{h=1}^{h^{🟉}}P_{h}\left(HMI\right)+P_{R}. \end{array}$$

In this case, the analyst only needs to evaluate $P_{h}\left(HMI\right)$ for $h=1,\ldots ,h^{🟉}$. The procedures to determine $h^{🟉}$ and the corresponding $P_{R}$ are described in [45,46]. These papers presents reasonable values for $h^{🟉}$ in the context of the historical data of single satellite failure. They do not consider situations with large numbers of faulty measurements due to spoofing, non-line-of-sight signals, etc.

### 5.1. Hypothesis Probabilities

Let $P(H_{h})$ denote the probability of one of the *h*-fault scenarios occurring. Usually the probabilities of occurrence of all fault hypotheses $H_{h,i}$ are assumed to be equal: $P\left(H_{h,i}\right)=P\left(H_{h,j}\right)for1\leq i,\;j\leq M_{h}.$ Then, the probability of occurrence of the *h*-fault scenario is

$$P\left(H_{h}\right)=\sum_{i=1}^{M_{h}}P\left(H_{h,i}\right)=M_{h}\;P\left(H_{h,1}\right)\mathrm{for}\;h=1,\cdots ,m.$$

The allocation of the nominal probabilities for each fault scenario is such that

(31) $$\begin{array}{c} \sum_{h=1}^{m}M_{h}\;P\left(H_{h,1}\right)=1-P_{0}, \end{array}$$

where $P_{0}$ is the probability of the fault-free scenario. The value of each $P(H_{h,1})$ is small and determined empirically. See, e.g., ref. [7].

### 5.2. Evaluating $P(HMI_{h,i}\;|\;H_{h,i})$

The probability of HMI under the *i*-th of the *h*-fault hypotheses is

$$\begin{array}{cc} P(HMI_{h,i}\;|\;H_{h,i}) & =P\left(∥\delta \hat{x}{∥}^{2}>L^{2},\;T_{r}<T^{🟉}\;|\;H_{h,i}\right)\\ \; & =\;P\left((∥\delta \hat{x}{∥}^{2}\;>\;L^{2})\;|\;\left(T_{r}\;<\;T^{🟉},\;H_{h,i}\right)\right)\;P\left(T_{r}\;<\;T^{🟉}\;|\;H_{h,i}\right). \end{array}$$

The failure mode slope, which is reviewed in Section 6, provides a means of predicting the probability that $P\left((∥\delta \hat{x}{∥}^{2}\;>\;L^{2})\;|\;\left(T_{r}\;<\;T^{🟉},\;H_{h,i}\right)\right)$ based on $T_{r}$, as defined in Equation (26).

## 6. Failure Mode Slope

The literature defines the *failure mode slope* $g_{f}$ as the ratio between the norm of the fault-induced bias of the estimate, $∥\delta x_{f}∥$ and the norm of fault-induced residual $∥r_{f}∥$:

(32) $$\begin{array}{c} g_{f}≜\frac{∥\delta x_{f}∥}{∥r_{f}∥}, \end{array}$$

as in refs. [4,7,35,43,47,48,49,50]. Equation (32) can be reorganized as:

(33) $$\begin{array}{c} ∥\delta x_{f}∥=g_{f}\;∥r_{f}∥, \end{array}$$

which shows that, for a given $g_{f}$, the norm of the fault-induced estimation bias grows proportionately with norm of the fault-induced residual. The largest estimation error $∥\delta x_{f}∥$ induced by the fault f that is not detectable for the residual test $T_{r}\leq T^{🟉}$ is:

(34) $$\begin{array}{c} ∥\delta x_{f}∥=g_{f}\;\sqrt{T^{🟉}}. \end{array}$$

When the maximum failure mode slope is bounded, $g_{f}\leq {\bar{g}}_{f}$, over a specified set of possible fault scenarios (e.g., $1\leq h\leq h^{🟉}$), then selecting $T^{🟉}$ as a function of $\frac{L^{2}}{{\bar{g}}_{f}^{2}}$ allows the RAIM designer to control the probability of HMI. If $T_{r}>T^{🟉}$ or $g_{f}>{\bar{g}}_{f}$ for the current set of available satellites, RAIM can declare the navigation solution to be unavailable [11,48].

Based on Equations (15) and (25), for any given fault direction $\vec{f}$, the failure mode slope can be computed as:

(35) $$\begin{array}{c} g_{f}=\frac{∥\Sigma^{-1}U_{1}^{⊤}\vec{f}{∥}^{2}}{∥U_{2}^{⊤}\;\vec{f}{∥}^{2}}=\frac{∥\Sigma^{-1}{a∥}^{2}}{{∥b∥}^{2}}, \end{array}$$

where the fault direction $\vec{f}$ defines the coordinate vectors a and b, as in Equation (17). The failure mode slope depends on the satellite constellation geometry (i.e., H through Σ, $U_{1}$ and $U_{2}$), and on fault direction through $\vec{f}$, but not on the fault magnitude.

## 7. Best and Worst-Case Faults

Equation (35) shows that it is the direction of the fault, not the magnitude, that is of interest. Because Σ is diagonal, Equation (35) is equivalent to

(36) $$\begin{array}{cc} g_{f} & ≐\frac{\sum_{i=1}^{n}{\left(\frac{a_{i}}{\alpha_{i}}\right)}^{2}}{\sum_{j=1}^{m-n}{\left(b_{j}\right)}^{2}}. \end{array}$$

where $\alpha_{i}$ are the singular values of H. Based on Equation (36), with a and b defined as in Equation (17), the following conclusions are straightforward:

**Best Case:**  For any fault that has ∥a∥=0 and ∥b∥=1, the fault direction $\vec{f}\in span\left(U_{2}\right)$. In this case, the numerator is zero and the failure mode slope $g_{f}=0.$ Physically, this means that the fault has absolutely no impact on the state estimate.
**Worst Case:**  For any fault that has ∥a∥=1 and ∥b∥=0, the fault direction $\vec{f}\in span\left(U_{1}\right)$. In this case, the denominator is zero and the failure mode slope $g_{f}=\infty .$ Physically this means that the fault has no impact on the residual. Therefore, the residual test cannot detect it.

Note that, in both cases, the fault direction is not unique. Rather, each case allows an uncountably infinite number of fault directions within a finite dimensional subspace of $R^{m}$.

Within the *worst-case* subspace, certain directions can still be considered worse than others. The problem of finding a unit vector a that maximizes the numerator (i.e., the estimation error for μ=1) is formulated as:

(37) $$\begin{array}{cc} a & =\arg\max_{a}\sum_{i=1}^{n}{\left(\frac{a_{i}}{\alpha_{i}}\right)}^{2},\;\mathrm{subject}\;\mathrm{to}\;\sum_{i=1}^{n}a_{i}^{2}=1\\ \; & =\arg\max_{{∥a∥}^{2}=1}\left(a^{⊤}\Sigma^{-2}a\right). \end{array}$$

The problem in Equation (37) is a constrained quadratic maximization problem as in (5), of which the solution is the eigenvector corresponding to the largest eigenvalue of $U_{1}\Sigma^{-2}U_{1}^{⊤}$.

Therefore, the worst-case fault direction that has the greatest impact on the estimate per unit change in fault magnitude is the vector ${\vec{f}}_{w}=u_{1}^{n}$, making the worst-case fault vector $f=\mu \;u_{1}^{n}$. Using Equation (17), the coefficient vector corresponding to this fault direction is:

(38) $$a_{w}=\mu U_{1}^{⊤}u_{1}^{n}=\mu \;e_{n},$$

where $e_{n}\in R^{n}$ is the *n*-th standard *n* dimensional unit direction vector. Using Equation (14), the corresponding mean-squared estimation error is:

(39) $$\begin{array}{cc} {∥\delta x∥}_{M}^{2} & =\mu^{2}\left(e_{n}^{⊤}\;\Sigma^{-2}e_{n}\right)+\kappa_{1}={\left(\frac{\mu }{\alpha_{n}}\right)}^{2}+\kappa_{1}. \end{array}$$

This shows that the growth in the estimation error caused by the fault $f=\mu \;u_{1}^{n}$ is directly proportional to the inverse of the smallest singular value of H and the residual vector is not affected.

This section discussed the best and worst-case fault directions in the general case. Typically, these worst-case faults have non-zero contributions from all sensors; therefore, they do not correspond to any specific fault scenario with h<m. When a fault has a non-zero component in the linear space spanned by $U_{2}$ (i.e., ∥b∥≠0), then $g_{f}$ is finite. The next section analyzes and determines the worst-case fault for general *h*-fault scenarios.

## 8. Single, Double, Multi-Measurement Faults

This section considers different *h*-fault scenarios. The failure mode slope of Equation (32) can be used as the metric for fault impact; therefore, the worst-case fault direction is defined as:

(40) $$\begin{array}{cc} {\vec{f}}_{w} & =\arg\max_{{∥f∥}^{2}=1}g_{f}\left(f\right)=\arg\max_{{∥f∥}^{2}=1}\frac{∥\Sigma^{-1}U_{1}^{⊤}\vec{f}{∥}^{2}}{∥U_{2}^{⊤}\;\vec{f}{∥}^{2}}. \end{array}$$

This problem will be solved for different *h*-fault scenarios, beginning with faults affecting single measurements (i.e., h=1), then two measurements (i.e., h=2), and finally culminating with a general discussion of multi-measurements faults (i.e., h≥1). For each scenario, the analysis determines conditions when there are faults that cause the failure mode slope to be infinite. When the failure mode slope is finite the worst-case fault direction is determined.

### 8.1. Single-Measurement Faults

Single-measurement faults have the form $f=\mu \;e_{j}$ for j=1,⋯,m. For each such single-measurement fault, Equation (40) becomes

(41) $$\begin{array}{cc} g_{f}\left(e_{j}\right) & =\frac{e_{j}^{⊤}\;U_{1}\;\Sigma^{-2}U_{1}^{⊤}e_{j}}{e_{j}^{⊤}\;U_{2}\;U_{2}^{⊤}\;e_{j}}=\frac{\sum_{i=1}^{n}{\left(\frac{u_{1}^{i(j)}}{\alpha_{i}}\right)}^{2}}{\sum_{i=1}^{m-n}{\left(u_{2}^{i(j)}\right)}^{2}}. \end{array}$$

where $u_{1}^{i(j)}$ is the *j*-th element of vector $u_{1}^{i}$ for i=1,…,n and $u_{2}^{i(j)}$ is the *j*-th element of vector $u_{2}^{i}$ for i=1,…,(m−n). For each *j*, the value of $g_{f}\left(e_{j}\right)$ can be determined. The one with the largest value determines the worst-case single sensor failure (i.e., fault direction).

In the single fault scenario, $g_{f}$ can be infinite if an entire row of $U_{2}$ is zero. For example, the rank 4 matrix H defined as:

$$H=\frac{1}{2}\left[\begin{array}{cccc} 1 & -1 & \sqrt{2} & 2\\ \sqrt{2} & \sqrt{2} & 0 & 2\\ -1 & 1 & \sqrt{2} & 2\\ \sqrt{2}-1 & \sqrt{2}+1 & \sqrt{2} & 2\\ 1 & -1 & \sqrt{2} & 2 \end{array}\right]\mathrm{has}\;U_{2}=\left[\begin{array}{c} -\sqrt{2}\\ \;\;0\\ \;\;0\\ \;\;0\\ \;\;\sqrt{2} \end{array}\right],$$

which means that a single-sensor fault on any one of measurements 2, 3, or 4 cannot be detected by the residual (or parity) test statistic.

### 8.2. Double-Measurement Faults

For a double-measurement fault, two of the measurements are affected, not necessarily to the same extent. The fault is a linear combination of the individual measurement directions:

(42) $$f_{2}=\mu \left(s_{j}\;e_{j}+s_{k}\;e_{k}\right)=\mu \;e_{jk},$$

where, for j≠k, $e_{jk}≜\left(s_{j}\;e_{j}+s_{k}\;e_{k}\right)$ is the overall fault direction, and $s_{j},s_{k}\in \left[-1,1\right]$ are scaling coefficients such that $∥e_{jk}{∥}^{2}=s_{j}^{2}+s_{k}^{2}=1$.

The fault direction vector $e_{jk}$ can be written as:

(43) $$\begin{array}{c} e_{jk}=D_{jk}\;s, \end{array}$$

where $s\in R^{2}$, and $D_{jk}\in R^{m\times 2}$ are a binary matrix of which the only non-zero elements are in the *j*-th and *k*-th positions of the two columns, respectively, i.e.:

(44) $$\begin{array}{c} D_{jk}=\left[\begin{array}{cc} | & |\\ e_{j} & e_{k}\\ | & | \end{array}\right]. \end{array}$$

For each such two-fault mode, Equation (40) becomes

(45) $$\begin{array}{cc} g_{f}\left(e_{jk}\right)\; & =\frac{e_{jk}^{⊤}\;U_{1}\;\Sigma^{-1}U_{1}^{⊤}e_{jk}}{e_{jk}^{⊤}\;U_{2}\;U_{2}^{⊤}\;e_{jk}}\;\mathrm{for}∥e_{jk}∥\neq 0\\ \; & =\frac{s^{⊤}\Gamma_{jk}\;s}{s^{⊤}\;\Delta_{jk}\;s}\mathrm{for}∥s∥\neq 0, \end{array}$$

with $\Delta_{jk}=D_{jk}^{⊤}Q\;D_{jk}\in R^{2\times 2}$, $Q=U_{2}\;U_{2}^{⊤}$, and $\Gamma_{jk}=D_{jk}^{⊤}U_{1}\;\Sigma^{-2}\;U_{1}^{⊤}\;D_{jk}\in R^{2\times 2}.$ Given these definitions,

$$\begin{array}{ccc} \Gamma_{jk} & =\left[\begin{array}{cc} \sum_{i=1}^{n}\frac{{\left(u_{1}^{i(j)}\right)}^{2}}{\alpha_{i}^{2}}\; & \sum_{i=1}^{n}\frac{u_{1}^{i(j)}u_{1}^{i(k)}}{\alpha_{i}^{2}}\\ \sum_{i=1}^{n}\frac{u_{1}^{i(j)}u_{1}^{i(k)}}{\alpha_{i}^{2}}\; & \sum_{i=1}^{n}\frac{{\left(u_{1}^{i(k)}\right)}^{2}}{\alpha_{i}^{2}} \end{array}\right]\;\mathrm{and}\;\Delta_{jk} & =\left[\begin{array}{cc} \sum_{i=1}^{m-n}{\left(u_{2}^{i(j)}\right)}^{2}\; & \sum_{i=1}^{m-n}u_{2}^{i(j)}u_{2}^{i(k)}\\ \sum_{i=1}^{m-n}u_{2}^{i(j)}u_{2}^{i(k)}\; & \sum_{i=1}^{m-n}{\left(u_{2}^{i(k)}\right)}^{2} \end{array}\right]. \end{array}$$

Both $\Gamma_{jk}$ and $\Delta_{jk}$ are symmetric and, at least, positive semi-definite.

The failure mode slope for each pair (j,k) is determined by the value of s that maximizes $g_{f}\left(e_{jk}\right)$, as defined in Equation (45). This optimization problem is:

(46) $$\begin{array}{cc} s_{jk}^{*} & =\arg\max_{s\neq 0}\;\frac{s^{⊤}\Gamma_{jk}\;s}{s^{⊤}\Delta_{jk}\;s}. \end{array}$$

When $\Delta_{jk}$ is positive definite (i.e., nonsingular and invertible), then it has a square root matrix, denoted as $\Delta_{jk}^{\frac{1}{2}}$, which is, itself, positive definite and symmetric [42], and $\Delta_{jk}$ can be expressed as $\Delta_{jk}=\Delta_{jk}^{\frac{1}{2}}\Delta_{jk}^{\frac{1}{2}}$. As discussed in [35], the problem in Equation (46) is equivalent to

(47) $$\begin{array}{cc} s_{jk}^{*} & =\arg\max_{s\neq 0}\;\frac{s^{⊤}\Delta_{jk}^{\frac{1}{2}}\Delta_{jk}^{-\frac{1}{2}}\Gamma_{jk}\;\Delta_{jk}^{-\frac{1}{2}}\Delta_{jk}^{\frac{1}{2}}s}{s^{⊤}\Delta_{jk}^{\frac{1}{2}}\Delta_{jk}^{\frac{1}{2}}\;s}. \end{array}$$

Letting $q=\Delta_{jk}^{\frac{1}{2}}\;s$, the problem becomes:

(48) $$\begin{array}{c} \begin{array}{c} q_{jk}^{*}=\arg\max_{q\neq 0}\;\frac{q^{⊤}\Psi_{jk}\;q}{q^{⊤}\;q}, \end{array} \end{array}$$

where $\Psi_{jk}=\Delta_{jk}^{-\frac{1}{2}}\;\Gamma_{jk}\;\;\Delta_{jk}^{-\frac{1}{2}}\in R^{2\times 2}$. The problem in (48) is an eigenvalue problem of which the solution $q_{jk}^{*}$ is the eigenvector corresponding to the largest eigenvalue, denoted as $\lambda_{jk}^{*}$, of $\Psi_{jk}$. Therefore, $s^{*}=\Delta^{-\frac{1}{2}}\;q^{*}$ and $g_{f}^{*}=\lambda_{jk}^{*}$.

Each pair of satellites yields a value for $\lambda_{jk}^{*}$. There are m2 possible combinations that must be evaluated to quantify the worst-case impact of double-measurement faults on the estimation error and solution integrity.

### 8.3. Multi-Measurement Faults

This section generalizes the approach of Section 8.2 to the general case of *h* sensor failures.

A fault that affects *h* measurements, for 1≤h≤m, can be written as:

(49) $$\begin{array}{c} f_{h}=\mu \;e_{\pi }, \end{array}$$

where π denotes a permutation of the set of integers between 1 and *m* that has cardinality |π|=h. The fault direction vector $e_{\pi }$ can be written as:

(50) $$\begin{array}{c} e_{\pi }=D_{\pi }\;s, \end{array}$$

where $∥e_{\pi }{∥}^{2}=1$ and $s\in R^{h}$ with ${∥s∥}^{2}=1$. The matrix $D_{\pi }\in R^{m\times h}$ is as defined in (44), but with *h* unique columns.

For the *h*-fault scenario, the $g_{f}\left(f\right)$ in Equation (40) is equivalent to

(51) $$\begin{array}{cc} g_{f}\left(e_{\pi }\right)\; & =\frac{e_{\pi }^{⊤}\;U_{1}\;\Sigma^{-2}U_{1}^{⊤}e_{\pi }}{e_{\pi }^{⊤}\;U_{2}\;U_{2}^{⊤}\;e_{\pi }}for∥e_{\pi }∥\neq 0 \end{array}$$

(52) $$\begin{array}{cc} \; & =\frac{s^{⊤}\Gamma_{\pi }\;s}{s^{⊤}\;\Delta_{\pi }\;s}for∥s∥\neq 0. \end{array}$$

Letting $\pi_{j}$ denote the *j*-th element of π for j∈{1,…,h} and defining $\Phi =U_{1}\;\Sigma^{-2}\;U_{1}^{⊤}$, the matrix $\Gamma_{\pi }=D_{\pi }^{⊤}\;\Phi \;D_{\pi }\in R^{h\times h}$ has the form:

$$\begin{array}{c} \Gamma_{\pi }=\left[\begin{array}{cccc} \sum_{i=1}^{n}{\left(\frac{u_{1}^{i,\pi_{1}}}{\alpha_{i}}\right)}^{2} & \sum_{i=1}^{n}\frac{u_{1}^{i,\pi_{1}}u_{1}^{i,\pi_{2}}}{\alpha_{i}^{2}}\; & \cdots & \sum_{i=1}^{n}\frac{u_{1}^{i,\pi_{1}}u_{1}^{i,\pi_{h}}}{\alpha_{i}^{2}}\\ \sum_{i=1}^{n}\frac{u_{1}^{i,\pi_{2}}u_{1}^{i,\pi_{1}}}{\alpha_{i}^{2}} & \sum_{i=1}^{n}{\left(\frac{u_{1}^{i,\pi_{2}}}{\alpha_{i}}\right)}^{2}\; & \cdots & \sum_{i=1}^{n}\frac{u_{1}^{i,\pi_{2}}u_{1}^{i,\pi_{h}}}{\alpha_{i}^{2}}\\ \vdots & \vdots & \ddots & \vdots \\ \sum_{i=1}^{n}\frac{u_{1}^{i,\pi_{h}}u_{1}^{i,\pi_{1}}}{\alpha_{i}^{2}} & \sum_{i=1}^{n}\frac{u_{1}^{i,\pi_{h}}u_{1}^{i,\pi_{2}}}{\alpha_{i}^{2}}\; & \cdots & \sum_{i=1}^{n}{\left(\frac{u_{1}^{i,\pi_{h}}}{\alpha_{i}}\right)}^{2} \end{array}\right]. \end{array}$$

where $u_{1}^{i,\pi_{j}}$ denotes the element in row *i* and column $\pi_{j}$ of $U_{1}$ (similarly for $u_{2}^{i,\pi_{j}}$). The matrix $\Delta_{\pi }=D_{\pi }^{⊤}\;Q\;D_{\pi }\in R^{h\times h}$ has the form:

$$\begin{array}{c} \Delta_{\pi }=\left[\begin{array}{cccc} \sum_{i=1}^{m-n}{\left(u_{2}^{i,\pi_{1}}\right)}^{2} & \sum_{i=1}^{m-n}u_{2}^{i,\pi_{1}}u_{2}^{i,\pi_{2}}\; & \cdots & \sum_{i=1}^{m-n}u_{2}^{i,\pi_{1}}u_{2}^{i,\pi_{h}}\\ \sum_{i=1}^{m-n}u_{2}^{i,\pi_{2}}u_{2}^{i,\pi_{1}} & \sum_{i=1}^{m-n}{\left(u_{2}^{i,\pi_{2}}\right)}^{2}\; & \cdots & \sum_{i=1}^{m-n}u_{2}^{i,\pi_{2}}u_{2}^{i,\pi_{h}}\\ \vdots & \vdots & \ddots & \vdots \\ \sum_{i=1}^{m-n}u_{2}^{i,\pi_{h}}u_{2}^{i,\pi_{1}} & \sum_{i=1}^{m-n}u_{2}^{i,\pi_{h}}u_{2}^{i,\pi_{2}}\; & \cdots & \sum_{i=1}^{m-n}{\left(u_{2}^{i,\pi_{h}}\right)}^{2} \end{array}\right]. \end{array}$$

The matrix $\Delta_{\pi }$ is singular if any vector in the span of $D_{\pi }$ lies entirely in the span of $U_{1}$ or if at least one of the rows of $U_{2}$ is zero. Additionally, Section S4 of the Supplementary Materials [41] shows that $\Delta_{\pi }$ is singular whenever h>m−n but does not say anything about the case h≤(m−n).

As was conducted to convert from Equation (46) to (48), the square root of $\Delta_{\pi }$ defines $\Psi_{\pi }=\Delta_{\pi }^{-\frac{1}{2}}\;\Gamma_{\pi }\;\;\Delta_{\pi }^{-\frac{1}{2}}\in R^{h\times h}$, where $q=\Delta_{\pi }^{\frac{1}{2}}\;s$. The worst-case fault direction problem from Equation (40) for the *h*-fault scenario is equivalent to:

(53) $$\begin{array}{c} \begin{array}{c} q_{\pi }^{*}=\arg\max_{q\neq 0}\;\frac{q^{⊤}\Psi_{\pi }\;q}{q^{⊤}\;q}. \end{array} \end{array}$$

For some values of *h*, $\Delta_{\pi }$ can be singular for some π. Therefore, there are two cases to be considered: $\Delta_{\pi }$ is singular and $\Delta_{\pi }$ is nonsingular.

When $\Delta_{\pi }$ is nonsingular, the solution to Equation (53) is the eigenvector corresponding to the largest eigenvalue $\lambda_{\pi }^{*}$ of $\Psi_{\pi }$. Then, $s^{*}=\Delta_{\pi }^{-\frac{1}{2}}\;q_{\pi }^{*}$ and the worst-case fault direction is:

(54) $$\begin{array}{c} {\vec{f}}_{w}=D_{\pi }\;\Delta_{\pi }^{-\frac{1}{2}}\;q_{\pi }^{*}. \end{array}$$

The failure mode slope $g_{f}^{*}=\lambda_{\pi }^{*}$ is finite.

When $\Delta_{\pi }$ is singular, the matrix $\Delta_{\pi }^{-\frac{1}{2}}$ does not exist, so the problem in Equation (53) cannot be formulated and solved. This case is discussed in Section 8.4.

### 8.4. Undetectable Faults

This section further discusses the case that $\Delta_{\pi }$ is singular. Let *q* denote the number of zero eigenvalues of $\Delta_{\pi }$, with 0<q<h. Consider an eigendecomposition $\Delta_{\pi }=W\;A\;W^{⊤}$, where $W=\left[w_{1},\cdots ,w_{q},\cdots ,w_{h}\right]\in R^{h\times h}$ and $A=\mathrm{diag}\left(\lambda_{1},\cdots ,\lambda_{h}\right)\in R^{h\times h}$, with ${\left\{\lambda_{i}\right\}}_{i=1,\cdots ,q}=0$. The eigenvectors corresponding to the zero eigenvalues span a linear subspace of which the basis is ${\left\{w_{i}\right\}}_{i=1,\cdots ,q}$. For s in this linear subspace:

$$\begin{array}{c} s=\sum_{i=1}^{q}c_{i}\;w_{i}=W_{q}\;c, \end{array}$$

where $W_{q}=\left[w_{1},\cdots ,w_{q}\right]\in R^{h\times q}$ and $c\in R^{q}$, with ${∥c∥}^{2}=1$. Any s in this subspace will be in the null space of $\Delta_{\pi }$, meaning that $r_{f}=0$. Therefore, $g_{f}=\infty$. The fault-induced estimation error is $\delta x_{f}=\Sigma^{-1}U_{1}^{⊤}D_{\pi }W_{q}\;c$, with norm

(55) $$\begin{array}{cc} ∥\delta x_{f}{∥}^{2} & =c^{⊤}\;\Upsilon \;c, \end{array}$$

where $\Upsilon =W_{q}^{⊤}D_{\pi }^{⊤}U_{1}\Sigma^{-2}U_{1}^{⊤}D_{\pi }W_{q}$ is symmetric and positive semi-definite. The vector that maximizes the quadratic form in Equation (55) is:

(56) $$\begin{array}{c} c^{*}=\arg\max_{{∥c∥}^{2}=1}c^{⊤}\Upsilon \;c. \end{array}$$

Equation (56) is an eigenvalue problem; therefore, $c^{*}$ is the eigenvector associated with the largest eigenvalue $\upsilon^{*}$ of Υ. Of all the undetectable fault directions, the fault direction that causes the largest estimation error is

(57) $$\begin{array}{c} {\vec{f}}_{w}=D_{\pi }W_{q}\;c^{*}; \end{array}$$

and the worst-case fault-induced estimation error (per unit fault) is: $\max_{c}(∥\delta x_{f}{∥}^{2})=\upsilon^{*}.$

### 8.5. Effect of Number of Faults on Failure Mode Slope

Whether $g_{f}$ is finite (i.e., $\Delta_{\pi }$ is singular) depends on (1) how many measurements have faults, *h*, and (2) the structure of the measurement matrix H. The discussion below summarizes how the failure mode slope changes with the number of faults *h*.

(a) When 1≤h≤(m−n), $\Delta_{\pi }$ can be either singular or nonsingular. When nonsingular, $\mathrm{rank}(\Delta_{\pi })=h$, the worst-case fault direction is given by Equation (54), and $g_{f}<\infty$. When singular, $\mathrm{rank}(\Delta_{\pi })<h$, the worst-case fault direction is given by Equation (57) and the failure mode slope is unbounded, i.e., $g_{f}=\infty$.
(b) As *h* increases toward (m−n), the numerator in Equation (40) is bounded above by the squared reciprocal of the smallest singular value (i.e., $\frac{1}{\alpha_{n}^{2}}$), while the (worst-case) denominator can decrease toward zero, which causes the failure mode slope to increase toward infinity.
(c) When h>(m−n), $\Delta_{\pi }$ is singular. Therefore, there is at least one fault direction, as defined in Equation (57), that will make $∥r_{f}{∥}^{2}=0$. As a result, $g_{f}=\infty$.
(d) For h=m, $D_{\pi }=I_{m}$ and $\Delta_{\pi }=Q$. This $\Delta_{\pi }$ has (m−n) eigenvalues values that are one and *n* eigenvalues that are zero. The eigenvectors corresponding to the zero eigenvalues are in $\mathrm{span}(U_{1})$, which is the null space of $U_{2}$. As stated in Section 6, any fault in $\mathrm{span}(U_{1})$ is not detectable from the residual and has $g_{f}=\infty$. In particular, the worst-case fault direction is ${\vec{f}}_{w}=u_{1}^{n}$, which is undetectable and affects the state estimation error the most. This is the same solution as that in Equation (57).

## 9. Effect of Fault on Horizontal Position

The previous sections analyzed the general case for the full estimation vector x. In some applications, the analyst might only be interested in a portion of the estimation vector z that is linearly related to x. For example, in automotive transportation applications, there is often a focus on the horizontal position. In this case, the goal is to analyze how the worst-case fault affects the horizontal position, which satisfies $z=M\;{}_{E}^{T}R\;x$, where M and ${}_{E}^{T}R$ are defined in (3).

The failure mode slope associated with the horizontal position is defined as:

(58) $$\begin{array}{c} g_{f}≜\frac{∥\delta z_{f}{∥}^{2}}{∥r_{f}{∥}^{2}}\;. \end{array}$$

Because both y and $\hat{y}$ are unchanged despite the transformation on the estimation vector, the measurement residual r, defined in (19), remains unchanged. The horizontal position error is:

(59) $$\begin{array}{c} \delta z=M\;{}_{E}^{T}R\;V\;\Sigma^{-1}U_{1}^{⊤}\;\left(f+\eta \right), \end{array}$$

which makes the fault-induced and noise-dependent portions of the horizontal position error, respectively:

$$\begin{array}{c} \delta z_{f}=M\;{}_{E}^{T}R\;V\;\Sigma^{-1}U_{1}^{⊤}f\;\mathrm{and}\;\delta z_{\eta }=M\;{}_{E}^{T}R\;V\;\Sigma^{-1}U_{1}^{⊤}\eta . \end{array}$$

The worst-case fault direction for the horizontal position error can be found by the same methods used in the previous subsections. The only difference is that the matrix Φ used to compute Γ in Section 8.2 becomes:

(60) $$\begin{array}{c} \Phi =U_{1}\Sigma^{-1}V^{⊤}\;{}_{E}^{T}R^{⊤}M^{⊤}\;M\;{}_{E}^{T}R\;V\;\Sigma^{-1}U_{1}^{⊤}. \end{array}$$

The norm of the fault-induced horizontal position error is

(61) $$\begin{array}{c} ∥\delta z_{f}{∥}^{2}=f^{⊤}\Phi \;f, \end{array}$$

and the analysis in Section 8 for $g_{f}$ defined in (32) could be repeated with $g_{f}$ defined as in Equation (58).

## 10. Example Discussion

This section provides examples demonstrating selected topics from the paper in the context of GNSS applications.

### 10.1. Fixed Number of Measurements

Consider the measurement matrix H used in [43,49]:

(62) $$\begin{array}{c} H=\left[\begin{array}{cccc} \;\;0.7460266527 & -0.4689257437 & 0.4728137904 & 1\\ -0.8607445743 & -0.3446039300 & 0.3746557209 & 1\\ \;\;0.2109370676 & \;\;0.3502943374 & 0.9125784518 & 1\\ -0.0619331319 & -0.4967359072 & 0.8656891623 & 1\\ -0.7248969588 & \;\;0.4759681238 & 0.4979746422 & 1\\ -0.4009266538 & \;\;0.1274180997 & 0.9072058455 & 1 \end{array}\right]. \end{array}$$

The number of measurement is m=6. The number of variables to be estimated is n=4. The pseudorange noise vector η is considered to be zero-mean Gaussian with standard deviation $\sigma_{\eta }=3.30$ m, i.e., $\eta ∼N(0,\sigma_{\eta }^{2})$. The position vector portion of x is represented in a local tangent plane.

**Figure 1:**
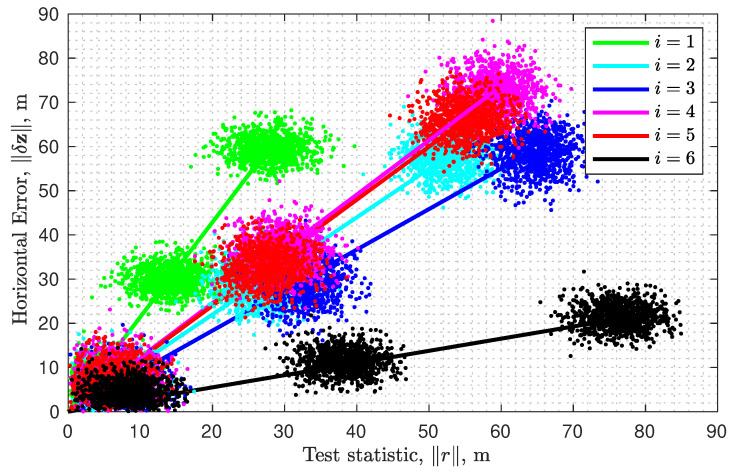
Illustration of failure mode slopes (straight lines) computed using (58) and points corresponding to measurements with worst-case faults of magnitudes μ=10,70,100.

For single-measurement faults, h=1, the fault is written as $f_{i}=\mu \;e_{i}\in R^{6}$, where $e_{i}$ is the *i*-th standard basis vector in the $R^{6}$ for i=1,⋯,6. The squared failure mode slope $g_{f}^{i}$ relating $\delta z_{f}$ to $r_{f}$ for each *i* is computed by Equation (41), as adapted for the horizontal case defined in Equation (58). The slope values are:

$$\sqrt{g_{f}^{i}}=\left\{2.144,\;1.099,\;0.917,\;1.228,\;1.199,\;0.275\right\},$$

for i=1,⋯,6. Figure 1 plots the horizontal position error norm versus the norm of the residual vector. This axes format matches that used in, e.g., refs. [10,11,43]. For each satellite, Figure 1 displays two items: (1) lines with slope $\sqrt{g_{f}^{i}}$, and (2) samples of ∥δz∥ computed for three values of μ and N=1000 instances of noise η. For each instance, the η,μ, and $e_{i}$ are used to construct a measurement vector y using (1), which is solved for ∥δz∥ using (59) and the residual-based test statistic ∥r∥ is computed using (24). Because η is a Gaussian random vector, simulating *N* measurements with the same $f_{i}$ produces a point cloud of (∥r∥,∥δz∥) values, each depicted by a dot. Each point cloud is an ellipsoid of which the center is the mean $\left(∥\delta r_{f}∥,\;∥\delta z_{f}∥\right)$ (for a given μ). For the smallest value of μ, the six clusters are very near the origin. As μ increases, the cluster corresponding to fault direction $e_{i}$ moves away from the origin along the line with slope $\sqrt{g_{f}^{i}}$, as the theory predicts.

The largest failure mode slope occurs for i=1 (i.e., green). This means that a fault on the measurements from satellite 1 will cause the largest change in the estimated vector per unit increase in $∥\delta r_{f}∥$.

This does not, however, mean that this fault will produce the largest estimated position error for a given value of μ, because its effect on both $∥\delta z_{f}∥$ and $∥\delta r_{f}∥$ can be small, while $g_{f}$ is their ratio. Table 1 shows the values of $∥\delta z_{f}∥$, $∥r_{f}∥$, and $g_{f}$ for μ=100. A fault on a measurement from satellite 4 produces the largest position error, but it also produces a proportionally larger test statistic. The value of μ affects both the numerator and denominator of $g_{f}$ but not their ratio and, hence, not the value of $g_{f}$.

Table 2 shows the worst-case squared failure mode slopes for different *h*-fault scenarios. In addition, it shows the fault-induced horizontal position error and residual squared norms, computed for μ=1. Due to the fact that the largest single measurement fault slopes in Table 1 correspond to measurements i=1 and i=4, one might assume that when h=2, the worst-case failure mode slope would occur when measurements from these two satellites are affected. This turns out not to be the case. The largest $g_{f}$ corresponds to faults on satellites 1 and 6 with coefficients $s=\left[\begin{array}{cc} 0.9352 & -0.3541 \end{array}\right]$ and the overall fault direction, as defined in (43).

### 10.2. Multi-Measurement Faults: Increasing *m*

Consider a matrix H consisting of m=20 GNSS measurements. This matrix is created using randomly selected elevation and azimuth angles, denoted by $E_{i}$ and $A_{i}$, respectively. To reflect practical GNSS situations, $E_{i}\in \left[15^{\circ },90^{\circ }\right]$ and $A_{i}\in \left[0^{\circ },360^{\circ }\right]$ for i=1,…,m. Each row of H represents a new measurement with $h_{i}=\left[\begin{array}{cc} 1_{i} & 1 \end{array}\right]$, where the unit line-of-sight vector is

$$1_{i}=\left[\begin{array}{ccc} \cos E_{i}\sin A_{i} & \cos E_{i}\cos A_{i} & \sin E_{i} \end{array}\right].$$

The matrix H is expressed as:

(63) $$\begin{array}{c} H={\left[h_{1}^{⊤},\cdots ,h_{m}^{⊤}\right]}^{⊤}. \end{array}$$

As *m* is increased, the first (m−1) rows remain the same, while one new row is added.

**Figure 2:**
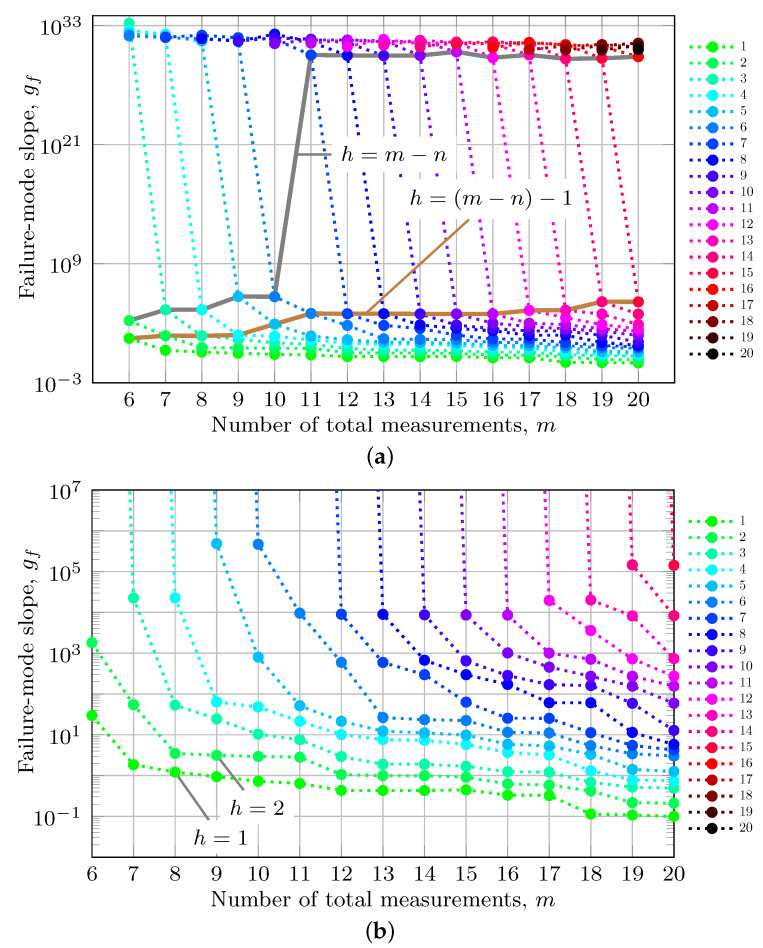
Maximum failure mode slope $g_{f}$ as a function of the number of measurements *m*. Each dotted curve is for a fixed value of *h*. (**a**) Full vertical scale graph of $g_{f}$. For m≥11, $g_{f}$ is numerically infinite for $h^{*}=\left(m-n\right)$. (**b**) Reduced vertical scale graph. For each *m*, $g_{f}$ increases with *h*. For each *h*, maximum $g_{f}$ decreases as *m* increases.

Using this H, for the first six fault scenarios (i.e., h=1,…,6), each column of Table 3 shows the worst-case $g_{f}$ for a fixed value of *h* as a function of *m*. Each column of Table 3 is graphed in Figure 2 as a dotted line of a unique color. The color code corresponding to different values of *h* is defined along the right side of the figure.

Note that, for each fixed *h* (i.e., any column), the value of $g_{f}$ decreases as *m* increases. Section 6 discussed that the detection threshold $T^{*}$ can be set as a function of the worst-case failure mode slope $g_{f}^{*}$. The fact that $g_{f}$ decreases for fixed *h* as *m* increases allows the designer to reduce integrity risk for the *h*-fault scenario by increasing the number of measurements used.

### 10.3. Multi-Measurement Faults: Increasing *h*

Each row of Table 3 shows the worst-case $g_{f}$ for a fixed value of *m* as a function of *h*. Note that for each fixed *m* (i.e., any row), the value of $g_{f}$ increases with *h*. The fact that $g_{f}$ increase for fixed *m* as *h* increases shows that integrity risk increases with higher *h*-fault scenarios.

For a given H, *m* is constant. The reason why the worst-case $g_{f}$ increases as a function of *h* is explained as follows. As *h* increases to $h^{\prime }=h+1$, the matrix $D_{\pi }$ gains an additional column and the vector s gains an additional row. This allows more flexibility in choosing the worst-case fault direction, which maximizes $g_{f}$. Whatever direction caused the worst-case fault for *h* is still a viable fault for $h^{\prime }$ (just set the coefficient for the new row of s to zero); however, the additional column of $D_{\pi }$ may allow the optimization defined in Section 8.3 to find a new fault direction such that $g_{f}\left(h^{\prime }\right)\geq g_{f}\left(h\right)$. The fact that the worst-case $g_{f}$ increases with *h* is shown by Table 2 for the specific H in Equation (62); each row in Table 3 for the H defined in Equation (63); and the colored dots at any fixed value of *m* in Figure 2.

**Figure 3:**
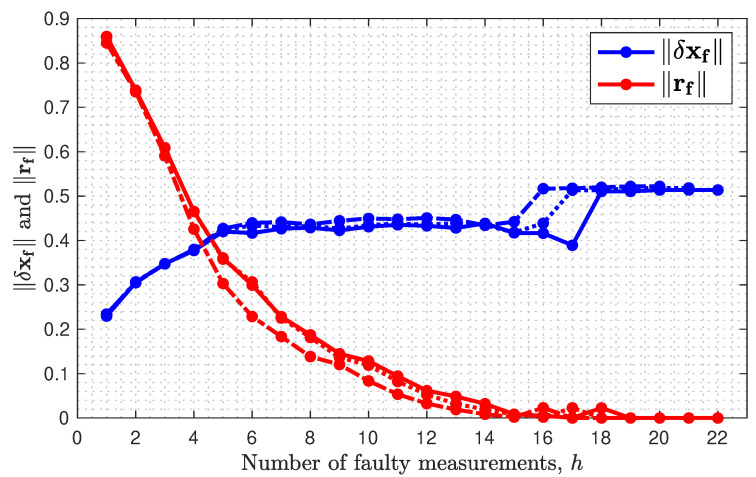
Norm of the fault-induced residual $∥r_{f}∥$ and estimation error $∥\delta x_{f}∥$ as a function of *h* for m=20 (dashed), m=21 (dotted), m=22 (solid).

Figure 3 separately graphs the numerator $∥x_{{\vec{f}}_{w}}∥$ (blue) and denominator $∥r_{{\vec{f}}_{w}}∥$ (red) of Equation (40) as a function of *h* for three values of *m* (dashed for m=20, dotted for m=21, and solid for m=22). For each *m*, as *h* increases, the value of $∥x_{{\vec{f}}_{w}}∥$ tends toward, but is bounded above by $\frac{1}{\alpha_{n}}$, where, for this example, $\alpha_{n}=1.938$ for m=22. The increase toward the upper bound is not monotonic. The worst-case fault ${\vec{f}}_{w}$ is defined based on the ratio $g_{f}$ defined in Equation (32). This ratio can increase even if the numerator decreases with *h*, as long as the denominator decreases at least a proportionate amount. For each *m*, as *h* increases, the value of $∥r_{{\vec{f}}_{w}}∥$ tends (not monotonically) toward zero. As the $∥r_{{\vec{f}}_{w}}∥$ approaches zero, $g_{f}$ approaches infinity. The fact that $g_{f}$ increases toward infinity is not due to $∥\delta x_{f}∥$ becoming very large but due to $∥r_{f}∥$ approaching zero.

### 10.4. How Does $g_{f}$ Become Infinite for h≤(m−n)?

Define $h^{*}$ as the value of *h* at which $\Delta_{\pi }$ becomes nonsingular for a given H. For $h\geq h^{*}$, there exist faults that do not affect the residual and, therefore, are undetectable from it for any threshold $T^{🟉}$.

Consider the H in Equation (63) that produced the results in Table 3. For m=6 and (m−n)=2, $g_{f}$ is finite for h=1 and 2 and infinite for h>2; therefore, $h^{*}=3$. For m=7 and (m−n)=3, $h^{*}=4$ and there are 3 finite values of $g_{f}$. For all values of *m*, there are, at most, (m−n) finite values of $g_{f}$ since $\Delta_{\pi }$ is singular for all h>(m−n). From Table 3, for m = 6 to 10, $h^{*}=\left(m-n\right)+1$, which is the known case [10].

As shown in Section 8.3, it is possible to have $h^{*}\leq \left(m-n\right)$. The gray line in Figure 2a corresponds to h=(m−n). When m≥11, there are only (m−n)−1 finite values of $g_{f}$ and, hence, $h^{*}=\left(m-n\right)$. The brown curve in Figure 2a corresponds to $g_{f}$ values for h=(m−n)−1. The general (though not absolute) trend of $g_{f}$ values on these curves is increasing with *m*, suggesting that as *m* increases the dimension of the subspace of undetectable faults may further increase, thus making $h^{*}=\left(m-n\right)-1$ as *m* increases.

This shows that increasing the number of measurements *m* results in a trade-off. For a given *h*, $g_{f}\left(m\right)$ decreases as *m* increases; however, $g_{f}\left(m-n\right)$ is non-decreasing with *m*, and $h^{🟉}\left(m\right)$ may become less than or equal to (m−n).

## 11. Conclusions

This paper analyzed the effect of measurement faults on the estimation error, measurement residual, and the failure mode slope in linear over-determined systems, such as GNSS. Of particular interest is the case where *m* is large because the number of GNSS measurements is increasing. The singular value decomposition provides formulas for the fault-induced estimation error and residual vector (see Equations (10) and (20), respectively) in terms of the orthogonal basis (i.e., $U_{1}$) for the range of H and the complement of the range space (i.e., $U_{2}$). This decomposition provides convenient formulas for the failure mode slope and worst-case fault in Equations (36) and (40). Using this decomposition, Section 7 provides $U_{1}$ as a basis for the *n* dimensional linear space of faults that are undetectable from the residual (or parity) vector and $U_{2}$ as the basis for the linear space of faults that affect the residual vector without affecting the estimate. For each fault scenario affecting *h* measurements, finding the worst-case fault is known to be an eigenvalue problem. Herein, this eigenvalue problem is written in terms of these two linear spaces, as in Equations (51) and (53). The detectability of any fault in the *h*-fault scenario from the residual vector depends on whether the range space of the matrix $D_{\pi }$, which is a permutation of *h* columns of the identity matrix, has a component in the linear space spanned by $U_{1}$. Building on this idea, it is clear from Equation (40) that, as *h* increases, the numerator is bounded above by the squared reciprocal of the smallest singular value (i.e., $\frac{1}{\alpha_{n}^{2}}$), while the (worst-case) denominator can decrease toward zero, which causes the failure mode slope to increase toward infinity. Alternatively, for a fixed *h*, as the number of measurements *m* increases, the dimension of the vector $∥r_{f}∥$ in the denominator increases, which tends to decrease the failure mode slope for *h*. Simulated GNSS examples are included to demonstrate the results derived herein.

## Figures and Tables

**Table 1:** Values for means of $∥\delta z_{f}∥$ and $∥r_{f}∥$, and $g_{f}$ for single-measurement faults, h=1, and μ=1 using H in (62).

*i*	$∥\delta z_{f}{∥}^{2}$	$∥r_{f}{∥}^{2}$	$g_{f}$
1	0.3496	0.0761	4.5955
2	0.3330	0.2755	1.2087
3	0.3479	0.4139	0.8405
4	0.5270	0.3496	1.5078
5	0.4367	0.3036	1.4382
6	0.0441	0.5813	0.0758

**Table 2:** Values of $∥\delta z_{f}∥$, $∥r_{f}∥$, and $g_{f}$ for worst-case fault modes when h=1,⋯,6 and μ=1 for H defined in Equation (62). The matrix Δ is singular for fault scenarios marked with an asterisk (${}^{*}$).

*h*	$∥\delta z_{f}{∥}^{2}$	$∥r_{f}{∥}^{2}$	$g_{f}$	Faulty Measurements
0	0	0	Undefined	[]
1	0.3496	0.0761	4.5955	[1]
2	0.3925	0.0085	46.2977	[1,6]
3 *	1.1456	0	*∞*	[3,4,5]
4 *	1.4856	0	*∞*	[2,3,4,5]
5 *	1.5028	0	*∞*	[1,2,3,4,5]
6 *	1.5254	0	*∞*	[1,2,3,4,5,6]

**Table 3:** Values of the worst-case $g_{f}$ for the first six fault scenarios. As *m* increases, the worst-case $g_{f}$ decreases *uniformly* for a fixed *h*. As *h* increases, the worst-case $g_{f}$ increases *uniformly* for a fixed *m*.

*m*	h=1	h=2	h=3	h=4	h=5	h=6
6	29.78	**1813.29**	*∞*	*∞*	*∞*	*∞*
7	1.85	54.32	**22,545.14**	*∞*	*∞*	*∞*
8	1.22	3.5	53.41	**22,729.26**	*∞*	*∞*
9	0.94	3.14	24.59	63.98	**4.86 × 10${}^{5}$**	*∞*
10	0.73	2.95	10.34	48.22	803.44	**4.69 × 10${}^{5}$**
11	0.64	2.83	7.59	21.47	51.57	9597.89
12	0.43	1.06	2.94	10.11	21.46	594.47
13	0.43	1	1.89	7.63	12.29	26.46
14	0.43	1	1.9	7.32	11.17	23.49
15	0.45	0.91	1.69	5.8	9.82	22.46
16	0.33	0.63	1.24	3.71	5.89	11.52
17	0.33	0.59	1.21	3.21	5.31	11.12
18	0.11	0.42	0.68	1.31	3.32	5.4
19	0.11	0.22	0.52	0.78	1.42	3.43
20	0.1	0.21	0.49	0.75	1.27	3.02

## Data Availability

No new data were created or analyzed in this study. Data sharing is not applicable to this article.

## Supplementary Materials

The following supporting information can be downloaded at: https://www.mdpi.com/article/10.3390/s23104947/s1.
