# Supplementary material for: RAIM and Failure Mode Slope: Effects of Increased Number of Measurements and Number of Faults
Source: Sensors (Basel). 2023 May 21;23(10):4947. doi: 10.3390/s23104947 (PMC10221399; doi:10.3390/s23104947)
Supplement: Supplementary file 1 [file sensors-23-04947-s001.zip › sensors-2349844-supplementary.pdf]

Supplementary Materials: RAIM and Failure Mode Slope: Effects of Increased Number of Measurements and Number of Faults

This document contains mathematical proofs and derivations as supplementary materials to an article[1] submitted Sensors Journal.

S1. SVD Interpretations of Various Quantities.

This appendix uses the SVD to represent various useful quantities.

Measurement Effects. It is useful to consider the physical meaning of the SVD in this application. The measurement model of eqn. (1), where the SVD of $\mathbf{H}$ is given in eqn. (4), can be rewritten as:

$$\mathbf{y} = [\mathbf{U}_1, \mathbf{U}_2]\left[\begin{pmatrix}\boldsymbol{\Sigma}\\ \mathbf{0}\end{pmatrix}\right]\mathbf{V}^\top \boldsymbol{x} + \boldsymbol{\eta} + \mathbf{f} \tag{S1}$$

$$= \sum_{i=1}^{4} \mathbf{u}_1^i\, \alpha_i\, (\mathbf{v}^i)^\top \boldsymbol{x} + \boldsymbol{\eta} + \mathbf{f} \tag{S2}$$

$$= \sum_{i=1}^{4} \mathbf{u}_1^i\, \alpha_i\, c_i + \boldsymbol{\eta} + \mathbf{f} \tag{S3}$$

where for $i = 1, \ldots, n$,

$$\begin{aligned}
\mathbf{U}_1 &= \left[\mathbf{u}_1^1, \ldots, \mathbf{u}_1^n\right] \text{ for } \mathbf{u}_1^i \in \mathbb{R}^{m\times 1};\\
\mathbf{V} &= \left[\mathbf{v}^1, \ldots, \mathbf{v}^n\right] \text{ for } \mathbf{v}^i \in \mathbb{R}^{n\times 1}; \text{ and },\\
c_i &= (\mathbf{v}^i)^\top \boldsymbol{x} \text{ for } i = 1, \ldots, n.
\end{aligned}$$

Note that $\mathbf{U}_2$ as an orthogonal basis for the complement of the range space of $\mathbf{H}$ plays no role in determining the value of $\mathbf{y}$. The quantities c_i are the coordinates for the vector $\boldsymbol{x}$ with respect to the orthogonal basis for $\mathbb{R}^n$ defined by the columns of $\mathbf{V}$. Because $\mathbf{V}$ is unitary, $\|\boldsymbol{x}\| = \|\mathbf{c}\|$. Consider the following:

- The vector $\mathbf{v}^1$ defines the direction of $\boldsymbol{x} \in \mathbb{R}^n$ that affects $\mathbf{y}$ the most. A unit change of $\boldsymbol{x}$ in the direction of $\mathbf{v}^1$ causes a change in $\mathbf{y}$ of magnitude α_1 in the direction $\mathbf{u}_1^1$.
- The vector $\mathbf{v}^4$ defines the direction of $\boldsymbol{x} \in \mathbb{R}^n$ that affects $\mathbf{y}$ the least. A unit change of $\boldsymbol{x}$ in the direction of $\mathbf{v}^4$ causes a change in $\mathbf{y}$ of magnitude α_4 in the direction $\mathbf{u}_1^4$.

Similar discussion holds for $\mathbf{v}^2$ and $\mathbf{v}^3$.

Representations of Useful Quantities. Using the SVD decomposition of (4), we have

$$\begin{aligned}
(\mathbf{H}^\top\mathbf{H})^{-1} &= \left(\mathbf{V}[\boldsymbol{\Sigma}\quad \mathbf{0}]\mathbf{U}^\top\mathbf{U}\begin{bmatrix}\boldsymbol{\Sigma}\\ \mathbf{0}\end{bmatrix}\mathbf{V}^\top\right)^{-1}\\
&= \left(\mathbf{V}[\boldsymbol{\Sigma}\quad \mathbf{0}]\begin{bmatrix}\boldsymbol{\Sigma}\\ \mathbf{0}\end{bmatrix}\mathbf{V}^\top\right)^{-1}\\
&= \left(\mathbf{V}\boldsymbol{\Sigma}^2\mathbf{V}^\top\right)^{-1}\\
&= \mathbf{V}\boldsymbol{\Sigma}^{-2}\mathbf{V}^\top.
\end{aligned} \tag{S4}$$

[1] Equations and other quantities from the main article may be referenced within this document without being explicitly restated.

Then,

$$\begin{aligned}\mathbf{H}^* &= (\mathbf{H}^\top \mathbf{H})^{-1}\mathbf{H}^\top \\ &= \mathbf{V}\boldsymbol{\Sigma}^{-2}\mathbf{V}^\top \mathbf{V}\begin{bmatrix}\boldsymbol{\Sigma} & \mathbf{0}\end{bmatrix}\mathbf{U}^\top \\ &= \mathbf{V}\begin{bmatrix}\boldsymbol{\Sigma}^{-1} & \mathbf{0}\end{bmatrix}\mathbf{U}^\top \\ &= \mathbf{V}\boldsymbol{\Sigma}^{-1}\mathbf{U}_1^\top,\end{aligned} \tag{S5}$$

which leads to

$$\begin{aligned}(\mathbf{H}^*)^\top(\mathbf{H}^*) &= \mathbf{U}_1\boldsymbol{\Sigma}^{-1}\mathbf{V}^\top\mathbf{V}\boldsymbol{\Sigma}^{-1}\mathbf{U}^\top \\ &= \mathbf{U}_1\boldsymbol{\Sigma}^{-1}\,\mathbf{I}_n\,\boldsymbol{\Sigma}^{-1}\mathbf{U}^\top \\ &= \mathbf{U}_1\boldsymbol{\Sigma}^{-2}\mathbf{U}_1^\top.\end{aligned} \tag{S6}$$

The matrix $(\mathbf{H}^*)^\top(\mathbf{H}^*) \in \mathbb{R}^{m\times m}$ is positive semi-definite and symmetric.

Worst-Case Faults. Using the properties of the matrices in the SVD, eqn. (7) can be expressed as

$$\hat{\boldsymbol{x}} = \sum_{i=1}^{4} \mathbf{v}^i\,\alpha_i^{-1}\,(\mathbf{u}_1^i)^\top\,\mathbf{y}. \tag{S7}$$

Because α_4 is smaller than the other α_i's, the output direction $\mathbf{u}_1^4$ has the greatest impact on the estimate. In the case of a worst-case fault, whose direction is $\mathbf{u}_1^4$ and magnitude is μ, eqn. (7) becomes

$$\hat{\boldsymbol{x}}_f = \sum_{i=1}^{4} \mathbf{v}^i\,\alpha_i^{-1}\,(\mathbf{u}_1^i)^\top\left(\bar{\mathbf{y}} + \mu\mathbf{u}_1^4\right) \tag{S8}$$

$$= \sum_{i=1}^{4}\left(\mathbf{v}^i\,\alpha_i^{-1}\,(\mathbf{u}_1^i)^\top\,\bar{\mathbf{y}}\right) + \mu\sum_{i=1}^{4}\left(\mathbf{v}^i\,\alpha_i^{-1}\,(\mathbf{u}_1^i)^\top\,\mathbf{u}_1^4\right) \tag{S9}$$

$$= \hat{\boldsymbol{x}} + \mu\,\mathbf{v}^4\,\alpha_4^{-1} \tag{S10}$$

$$= \hat{\boldsymbol{x}} + \xi_f, \tag{S11}$$

where $\bar{\mathbf{y}} \triangleq \mathbf{H}\,\boldsymbol{x} + \boldsymbol{\eta}$ is the fault-free measurement vector. Thus, the maximum added fault error magnitude is

$$\|\xi_f\| = \|\hat{\boldsymbol{x}}_f - \hat{\boldsymbol{x}}\| = \mu\,\alpha_4^{-1}.$$

Projection Matrices.

Lemma S1. *For the matrix* $\mathbf{P}$ *defined relative to eqn. (19):* $\mathrm{rank}(\mathbf{P}) = \mathrm{trace}(\mathbf{P}) = n$.

Proof: Substituting the SVD of $\mathbf{H}$ into the definition of $\mathbf{P}$ and simplifying yields:

$$\begin{aligned}\mathbf{P} &= \mathbf{H}\left(\mathbf{V}\,\boldsymbol{\Sigma}^2\,\mathbf{V}^\top\right)^{-1}\mathbf{H}^\top \\ &= \mathbf{U}\begin{bmatrix}\boldsymbol{\Sigma}\\ \mathbf{0}\end{bmatrix}\mathbf{V}^\top\left(\mathbf{V}\,\boldsymbol{\Sigma}^2\,\mathbf{V}^\top\right)^{-1}\mathbf{V}\begin{bmatrix}\boldsymbol{\Sigma} & \mathbf{0}\end{bmatrix}\mathbf{U}^\top \\ &= \mathbf{U}\begin{bmatrix}\mathbf{I}_n & \mathbf{0}\\ \mathbf{0} & \mathbf{0}_{m-n}\end{bmatrix}\mathbf{U}^\top \quad (S12) \\ &= \mathbf{U}_1\,\mathbf{U}_1^\top. \quad (S13)\end{aligned}$$

The right-hand side of eqn. (S12) provides an eigendecomposition of $\mathbf{P}$. The columns of $\mathbf{U}_1$ are eigenvectors corresponding to eigenvalues equal to one. The columns of $\mathbf{U}_2$ are eigenvectors corresponding to eigenvalues equal to zero. Therefore,

$$\text{trace}(\mathbf{P}) = \text{rank}(\mathbf{P}) = n.$$

■

Lemma S2. *For the matrix* $\mathbf{Q}$ *defined relative to eqn. (20):* $\text{rank}(\mathbf{Q}) = \text{trace}(\mathbf{Q}) = (m-n)$.

Proof: Following analysis similar to that leading to eqn. (S12) yields

$$\mathbf{Q} = \mathbf{U}\begin{bmatrix} \mathbf{0}_n & \mathbf{0} \\ \mathbf{0} & \mathbf{I}_{m-n} \end{bmatrix}\mathbf{U}^\top \tag{S14}$$

$$= \mathbf{U}_2\,\mathbf{U}_2^\top. \tag{S15}$$

The right-hand side of eqn. (S14) provides an eigendecomposition of $\mathbf{Q}$. Each column of $\mathbf{U}_1$ is an eigenvector of $\mathbf{Q}$ with eigenvalue equal to zero. Each column of $\mathbf{U}_2$ is an eigenvector of $\mathbf{Q}$ with eigenvalue equal to one. Therefore,

$$\text{trace}(\mathbf{Q}) = \text{rank}(\mathbf{Q}) = (m-n).$$

■

S2. Derivation of the Estimation MSE

The goal of this appendix is to derive the expressions in eqns. (14) and (15). Starting from eqn. (10), the MSE of $\delta\boldsymbol{x}$ can be express as

$$\begin{aligned} E\langle(\delta\boldsymbol{x})^\top(\delta\boldsymbol{x})\rangle &= E\langle(\delta\boldsymbol{x}_\eta + \delta\boldsymbol{x}_\mathbf{f})^\top(\delta\boldsymbol{x}_\eta + \delta\boldsymbol{x}_\mathbf{f})\rangle \\ &= E\langle(\delta\boldsymbol{x}_\mathbf{f})^\top(\delta\boldsymbol{x}_\mathbf{f})\rangle + E\langle(\delta\boldsymbol{x}_\eta)^\top(\delta\boldsymbol{x}_\eta)\rangle \\ &= \|\delta\boldsymbol{x}_\mathbf{f}\|^2 + \|\delta\boldsymbol{x}_\eta\|_M^2 \\ \|\delta\boldsymbol{x}\|_M^2 &= \|\delta\boldsymbol{x}_\mathbf{f}\|^2 + \kappa_1 \end{aligned} \tag{S16}$$

where $\kappa_1 \doteq \|\delta\boldsymbol{x}_\eta\|_M^2 = E\langle(\delta\boldsymbol{x}_\eta)^\top(\delta\boldsymbol{x}_\eta)\rangle$. This expression simplifies as follows:

$$\begin{aligned} \kappa_1 &= E\langle(\delta\boldsymbol{x}_\eta)^\top(\delta\boldsymbol{x}_\eta)\rangle \\ &= E\left\langle tr\left(\boldsymbol{\eta}^\top\,\mathbf{U}_1\,\boldsymbol{\Sigma}^{-1}\mathbf{V}^\top\mathbf{V}\,\boldsymbol{\Sigma}^{-1}\,\mathbf{U}_1^\top\boldsymbol{\eta}\right)\right\rangle \\ &= tr\left(\boldsymbol{\Sigma}^{-2}\,\mathbf{U}_1^\top E\left\langle\boldsymbol{\eta}\boldsymbol{\eta}^\top\right\rangle\mathbf{U}_1\right) \\ &= \sigma_\eta^2\,tr\left(\boldsymbol{\Sigma}^{-2}\right) \end{aligned} \tag{S17}$$

From (12), $\mathbf{C}_x = \sigma_\eta^2\,\mathbf{V}\boldsymbol{\Sigma}^{-2}\mathbf{V}^\top$, so that

$$\begin{aligned} \text{tr}(\mathbf{C}_x) &= \sigma_\eta^2\,\text{tr}\left(\mathbf{V}\boldsymbol{\Sigma}^{-2}\mathbf{V}^\top\right) = \sigma_\eta^2\,\text{tr}\left(\boldsymbol{\Sigma}^{-2}\mathbf{V}^\top\mathbf{V}\right) \\ &= \sigma_\eta^2\,\text{tr}\left(\boldsymbol{\Sigma}^{-2}\right). \end{aligned}$$

Therefore, $\kappa_1 = \text{tr}(\mathbf{C}_x)$.

S3. Derivation of the MSE of the Residual r

Starting from eqn. (20), the correlation matrix for $\mathbf{r}$ is

$$\begin{aligned}
E\langle \mathbf{r}\mathbf{r}^\top \rangle &= E\langle \mathbf{Q}(\mathbf{f}+\boldsymbol{\eta})(\mathbf{f}+\boldsymbol{\eta})^\top \mathbf{Q}^\top \rangle \\
&= E\langle \mathbf{Q}\,\mathbf{f}\,\mathbf{f}^\top \mathbf{Q}^\top \rangle + \mathbf{E}\langle \mathbf{Q}\,\mathbf{f}\text{[illegible]}^\top \mathbf{Q}^\top \rangle \\
&\quad + E\langle \mathbf{Q}\,\boldsymbol{\eta}\,\mathbf{f}^\top \mathbf{Q}^\top \rangle + \mathbf{E}\langle \mathbf{Q}\text{[illegible]}^\top \mathbf{Q}^\top \rangle \\
&= \mathbf{Q}\,\mathbf{f}\,\mathbf{f}^\top \mathbf{Q}^\top + \mathbf{Q}\,\sigma_\eta^2\,\mathbf{I}_m \mathbf{Q}^\top \\
&= \mathbf{Q}\,\mathbf{f}\,\mathbf{f}^\top \mathbf{Q}^\top + \text{œ}^2_{\text{[illegible]}}\mathbf{Q}.
\end{aligned} \tag{S18}$$

The MSE for the residual $\mathbf{r}$ is:

$$\begin{aligned}
E\langle \mathbf{r}^\top \mathbf{r} \rangle &= E\langle \mathrm{tr}\big(\mathbf{r}^\top\,\mathbf{r}\big)\rangle = E\langle \mathrm{tr}\big(\mathbf{r}\mathbf{r}^\top\big)\rangle \\
&= \mathrm{E}\Big\langle \mathrm{tr}\big(\mathbf{Q}(\boldsymbol{\eta}+\mathbf{f})(\boldsymbol{\eta}+\mathbf{f})^\top \mathbf{Q}^\top\big)\Big\rangle \\
&= \mathrm{tr}\Big(\mathbf{Q}\,\mathbf{f}\,\mathbf{f}^\top \mathbf{Q}^\top + \sigma_\eta^2\,(\mathbf{Q}\,\mathbf{I}_m \mathbf{Q}^\top)\Big) \\
&= \mathrm{tr}(\mathbf{Q}\,\mathbf{f}\,\mathbf{f}^\top \mathbf{Q}^\top) + \sigma_\eta^2\,\mathrm{tr}(\mathbf{Q}^\top \mathbf{Q}) \\
&= \mathbf{f}^\top \mathbf{Q}^\top \mathbf{Q}\,\mathbf{f} + \sigma_\eta^2\,\mathrm{tr}(\mathbf{Q}) \\
\|\mathbf{r}\|_M^2 &= \|\mathbf{U}_2^\top\,\mathbf{f}\|^2 + \sigma_\eta^2\,(m-n)
\end{aligned} \tag{S19}$$

S4. Invertibility Conditions

This section provides the conditions on the number of faults such that the matrix $\boldsymbol{\Delta}_\pi$ in (50) will be non-invertible, i.e. singular. Recall that

$$\boldsymbol{\Delta}_\pi = \mathbf{D}_\pi^\top\,\mathbf{Q}\,\mathbf{D}_\pi,$$

where $\boldsymbol{\Delta}_\pi \in \mathbb{R}^{h\times h}$, $\mathbf{Q} \in \mathbb{R}^{m\times m}$ with orthonormal columns, and $\mathbf{D}_\pi \in \mathbb{R}^{m\times h}$ is a binary matrix with exactly one non-zero element per column. The rank of matrices $\mathbf{D}_\pi$ and $\mathbf{Q}$ are:

$$\begin{aligned}
\mathrm{rank}(\mathbf{D}_\pi) &= h \\
\mathrm{rank}(\mathbf{Q}) &= m-n.
\end{aligned}$$

Claim: The matrix $\boldsymbol{\Delta}_\pi$ is not invertible if $h > m-n$.
Proof: The invertible matrix theorem[1] states that $\boldsymbol{\Delta}_\pi \in \mathbb{R}^{h\times h}$ is invertible if and only if it has full rank; which would require $\mathrm{rank}(\boldsymbol{\Delta}_\pi) = h$.

Because

$$\begin{aligned}
\mathbf{Q}^\top \mathbf{Q} = \mathbf{Q}\,\mathbf{Q} &= \mathbf{Q}, \\
\mathrm{rank}(\mathbf{A}^\top \mathbf{A}) = \mathrm{rank}(\mathbf{A}) &= \mathrm{rank}(\mathbf{A}^\top),\ \text{and} \\
\mathrm{rank}(\mathbf{A}\,\mathbf{B}) &\le \min(\mathrm{rank}(\mathbf{A}),\ \mathrm{rank}(\mathbf{B}))
\end{aligned} \tag{S20}$$

it is true that

$$\begin{aligned}
\mathrm{rank}(\boldsymbol{\Delta}_\pi) &= \mathrm{rank}\Big(\mathbf{D}_\pi^\top \mathbf{Q}\,\mathbf{D}_\pi\Big) \\
&= \mathrm{rank}\Big(\mathbf{D}_\pi^\top \mathbf{Q}^\top\,\mathbf{Q}\,\mathbf{D}_\pi\Big) \\
&= \mathrm{rank}\Big((\mathbf{Q}\,\mathbf{D}_\pi)^\top (\mathbf{Q}\,\mathbf{D}_\pi)\Big) \\
&= \mathrm{rank}(\mathbf{Q}\,\mathbf{D}_\pi) \\
&\le \min(\mathrm{rank}(\mathbf{Q}),\ \mathrm{rank}(\mathbf{D}_\pi)) \\
\mathrm{rank}(\boldsymbol{\Delta}_\pi) &\le \min(m-n,\ h)
\end{aligned}$$

Therefore, $\boldsymbol{\Delta}_\pi$ is not invertible when $h > (m - n)$. ■

The interpretation of this result is as follows. As stated in Section 6, any fault in $span(\mathbf{U}_1)$ is not detectable from the residual. When $h > (m - n)$, it is guaranteed that there exists a fault $\mathbf{f}$ in the n-dimensional $span(\mathbf{U}_1)$ will be in the h-dimensional $span(\mathbf{D}_\pi)$.

Note that even when $h \leq (m - n)$ it is possible for $\boldsymbol{\Delta}_\pi$ to be singular. An example is when any column of $\mathbf{D}_\pi$ is in $span(\mathbf{U}_1)$.

1. Horn, R.A.; Johnson, C.R. *Matrix Analysis*; Cambridge University Press, 1985.
